# An Event-Related fMRI Study of Phonological Verbal Working Memory in Schizophrenia

**DOI:** 10.1371/journal.pone.0012068

**Published:** 2010-08-11

**Authors:** Jejoong Kim, Natasha L. Matthews, Sohee Park

**Affiliations:** 1 Department of Psychology and the Center for Integrative and Cognitive Neuroscience, Vanderbilt University, Nashville, Tennessee, United States of America; 2 Department of Psychiatry, Vanderbilt University School of Medicine, Nashville, Tennessee, United States of America; 3 Department of Brain and Cognitive Sciences, Seoul National University, Seoul, Korea; 4 The Queensland Brain Institute, The University of Queensland, St Lucia, Queensland, Australia; University of Groningen, Netherlands

## Abstract

**Background:**

While much is known about the role of prefrontal cortex (PFC) in working memory (WM) deficits of schizophrenia, the nature of the relationship between cognitive components of WM and brain activation patterns remains unclear. We aimed to elucidate the neural correlates of the maintenance component of verbal WM by examining correct and error trials with event-related fMRI.

**Methodology/Findings:**

Twelve schizophrenia patients (SZ) and thirteen healthy control participants (CO) performed a phonological delayed-matching-to-sample-task in which a memory set of three nonsense words was presented, followed by a 6-seconds delay after which a probe nonsense word appeared. Participants decided whether the probe matched one of the targets, and rated the confidence of their decision. Blood-oxygen-level-dependent (BOLD) activity during WM maintenance was analyzed in relation to performance (correct/error) and confidence ratings. Frontal and parietal regions exhibited increased activation on correct trials for both groups. Correct and error trials were further segregated into true memory, false memory, guess, and true error trials. True memory trials were associated with increased bilateral activation of frontal and parietal regions in both groups but only CO showed deactivation in PFC. There was very little maintenance-related cortical activity during guess trials. False memory was associated with increased left frontal and parietal activation in both groups.

**Conclusion:**

These findings suggest that a wider network of frontal and parietal regions support WM maintenance in correct trials compared with error trials in both groups. Furthermore, a more extensive and dynamic pattern of recruitment of the frontal and parietal networks for true memory was observed in healthy controls compared with schizophrenia patients. These results underscore the value of parsing the sources of memory errors in fMRI studies because of the non-linear nature of the brain-behavior relationship, and suggest that group comparisons need to be interpreted in more specific behavioral contexts.

## Introduction

Working memory (WM) deficit in schizophrenia is a cardinal feature of the disorder and is a potential candidate for an endophenotypic marker [1]. WM is a limited-capacity, active short-term memory system that guides and controls behavior in context [2], [3]. A majority of patients with schizophrenia show stable WM deficits [4] across diverse paradigms, modalities and methods [5]. Impaired verbal WM predicts poor functional outcome [6] and WM deficits have become a major therapeutic target for pharmacological treatments. Therefore it has become increasingly important to understand and specify the reasons for this deficit.

Clear evidence exists for the central role of the dorsolateral prefrontal cortex (DLPFC) in WM and its regulation of higher cognitive functions in non-human primates [7]. Past studies using single cell recording revealed that maintenance of WM representations is coded by increased firing rate of cells in the principal sulcus (PS, Area 46) and this robust increase of prefrontal activity during WM maintenance is correlated with accuracy of the task performance [8]–[10]. Similarly, WM accuracy is correlated with increased DLPFC activation in healthy humans in neuroimaging studies [11]–[13]. However, numerous neuroimaging studies of WM have demonstrated task-related hypofrontality in schizophrenia patients [14], [15]. On the other hand, some studies have also observed hyperfrontality in schizophrenia [16], [17]. This discrepancy may arise from different WM loads across studies [18]. In healthy people DLPFC activity increases with WM load until the capacity of WM is exceeded at which point, it decreases [19], [20]. This relationship between WM load and DLPFC activity, often described as an inverted U, appears to be shifted in schizophrenia patients such that peak DLPFC activation is reached at a lower memory load compared with healthy controls. This hypothesis is supported by studies that demonstrate increased DLPFC activation in individuals with schizophrenia relative to controls for lower WM load [14], [16], [17] but reduced DLPFC activation with higher WM load [21], [22]. These findings have been interpreted as evidence for an inefficient WM system in schizophrenia such that they must “work harder” to maintain accuracy as WM load increases [14], [18], [22], [23].

One difficulty in interpreting these discrepant results is that very few studies have examined neural activity yoked to behavior on a trial-by-trial basis using an event-related design; the majority of fMRI studies of WM in schizophrenia have utilized block designed tasks that do not allow analyses of neural activation linked with specific type of responses.

Recently, Lee and colleagues [13] conducted an event-related fMRI and near-infrared spectroscopy (NIRS) study of spatial WM in schizophrenia to investigate prefrontal activation associated with correct and incorrect memory trials during WM maintenance. The rationale of this study follows from the known neural correlates of success and failure during WM tasks in non-human primates; the increased firing rates of PS cells are correlated with WM maintenance on the trials that the targets are remembered correctly but not on error trials [8]–[10]. Lee et al [13] observed increased prefrontal activation during WM maintenance on correct trials in both controls and patients. However, healthy controls recruited right frontal and parietal regions, consistent with a right hemisphere specialization for spatial processing [24]. On the other hand, schizophrenia patients showed a more bilateral frontoparietal activation pattern. Furthermore, they found that schizophrenia patients produced a large proportion of “false memory” errors (i.e. incorrect response with high confidence). Frontoparietal regions were recruited equally for false and correct memory trials, suggesting active maintenance of internal representation during the delay whether that representation was correctly or incorrectly encoded. This finding suggests that hyper or hypofrontality in schizophrenia may need to be re-interpreted. For example, hyperfrontality coupled with increased verbal WM errors in schizophrenia patients [16] is often interpreted in the context of general “inefficiency”. The concept of inefficiency could be further refined by distinguishing the case where there is unspecific increased neural activity versus the case where there is a specific increase in activity due to the maintenance of incorrectly encoded material. The former case would signify a true case of general inefficiency but the latter represents appropriate maintenance of incorrectly encoded stimulus. Both cases would look similar on the surface (i.e., hyperactivity coupled with WM errors). The crucial difference is that in the latter case, although the participant had an encoding error, the maintenance process itself is intact. It is possible that many WM errors made by persons with schizophrenia could arise because they maintain incorrectly encoded target representations. In this case, the problem would lie in the encoding process and not in the maintenance, and a general inefficiency hypothesis would not provide an optimal model.

The major goal of the present study was to elucidate the neural correlates of success and failure during verbal WM performance using an event-related design. Lee et al. [13] focused on spatial WM. In addition, they observed that schizophrenic patients tended to show both left and right frontal activity during spatial WM maintenance compared with the control participants, who showed a more right-lateralized network of activity during spatial WM maintenance. It would be important to ascertain if these findings generalize to the verbal domain.

**Figure 1:**
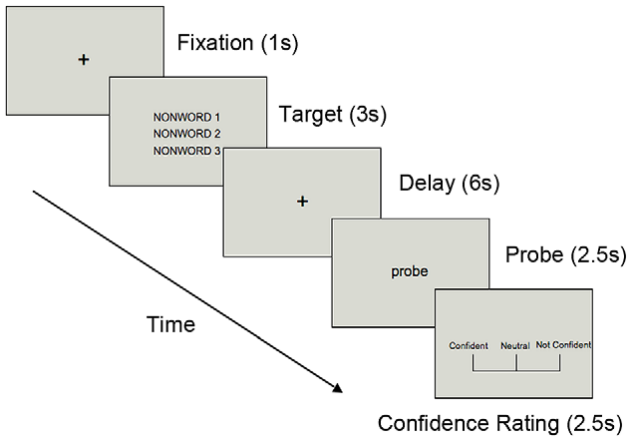
Procedure of the phonological verbal WM task

In the present experiment, we compared cortical activation in schizophrenia patients and healthy controls on a phonological delayed-matching-to-sample task (see Fig. 1) using an event-related design. We were specifically interested in examining neural activity associated with correct vs. error trials. In the delayed-matching-to-sample task, participants were asked to encode three nonsense words, followed by a 6-seconds delay period. Then a probe nonsense word was presented. Participants were asked to decide whether the probe word matched one of the three nonsense words from the encoding phase. Immediately after the recognition task, subjects were asked to rate the confidence of their recognition response. This procedure allowed us to separate correct and error trials based on the accuracy of their response, and to further divide correct and error trials according to the confidence ratings in order to examine hypothesized true memory vs. false memory trials. Considering the results from Lee et al. [13], we hypothesized that patients would show reduced frontal asymmetry corresponding to correct trials during the verbal WM task. Moreover, we hypothesized that neural activity corresponding to true correct and false memory trials would be very similar in SZ as well as in controls if during the delay period, the maintenance process is intact.

## Results

### Behavioral data

All significant tests are 2-tailed unless otherwise noted. We excluded trials with missing responses or missing confidence ratings. Mean number of excluded trials was 17.5 (SD = 19.7) in CO and 26.3 (SD = 16.4) in SZ. This difference was not statistically significant (t(23) = 1.20, p = 0.24).

Difference in mean overall % correct (82.6 (SD = 10.5) in CO; 76.8 (SD = 12.1) in SZ) was not statistically significant (t(23) = 1.27, p = 0.22, Cohen's *d* = 0.53), suggesting that this group of SZ did not show a significant overall deficit in verbal WM overall.

Correct trials were further segregated into “confident” and “not confident” trials. We categorized correct-and-confident trials as ‘*true correct memory*’ trials in which correct encoding and adequate maintenance are assumed to have taken place. *The number of true correct memory* trials was greater in CO than in SZ, with a large effect size (t(23) = 1.91, p = 0.03, 1-tailed, Cohen's *d* = 0.79). This suggests that SZ may be impaired in phonological verbal WM.

Correct-but-not-confident trials were hypothesized to be *guess* trials because the participants produced correct responses but had no idea if they were correct (i.e., they were guessing). The two groups did not differ in the number of guess trials (t(23) = −1.1, p = 0.28, Cohen's *d* = 0.46).

Among error trials, we examined ‘*false memory*’ trials in which subjects were wrong but nevertheless were highly confident of that they were right. In these trials, subjects were likely to have encoded incorrect stimuli and maintaining them in WM during the delay. Therefore, they are expected to be confident of their responses since they did remember, albeit incorrect items. Although SZ made more false memory errors than did controls but this difference was not statistically significant (t(23) = −1.30, p = 0.22, Cohen's *d* = 0.54).

The number of error trials with low confidence (true error) was miniscule and almost identical between the two groups. Behavioral results are summarized in Table 1.

**Table 1:** Summary of behavioral performance.

	SZ (n = 12)	CO (n = 13)	*t*	*p*	Effect size (Cohen's *d*)
Number of excluded trials	26.25 (16.43)^a^	17.46 (19.67)	−1.21	0.24	0.50
% Correct trials	76.84 (12.10)	82.56 (10.48)	1.27	0.11^b^	0.53
% True memory	56.29 (21.33)	69.55 (12.76)	1.91	0.03^b^	0.79
% Correct guess	20.55 (23.56)	13.01 (7.13)	−1.10	0.14^b^	0.46
% False memory	16.14 (12.88)	10.37 (9.07)	−1.30	0.11^b^	0.54
% True error	7.01 (6.61)	7.05 (6.61)	0.013	0.49^b^	0.005
% Confident trials	72.42 (27.87)	79.93 (11.42)	0.89	0.38	0.37

a Mean (standard deviation).

b 1-tailed.

### fMRI data

#### Correct trials

**Figure 2:**
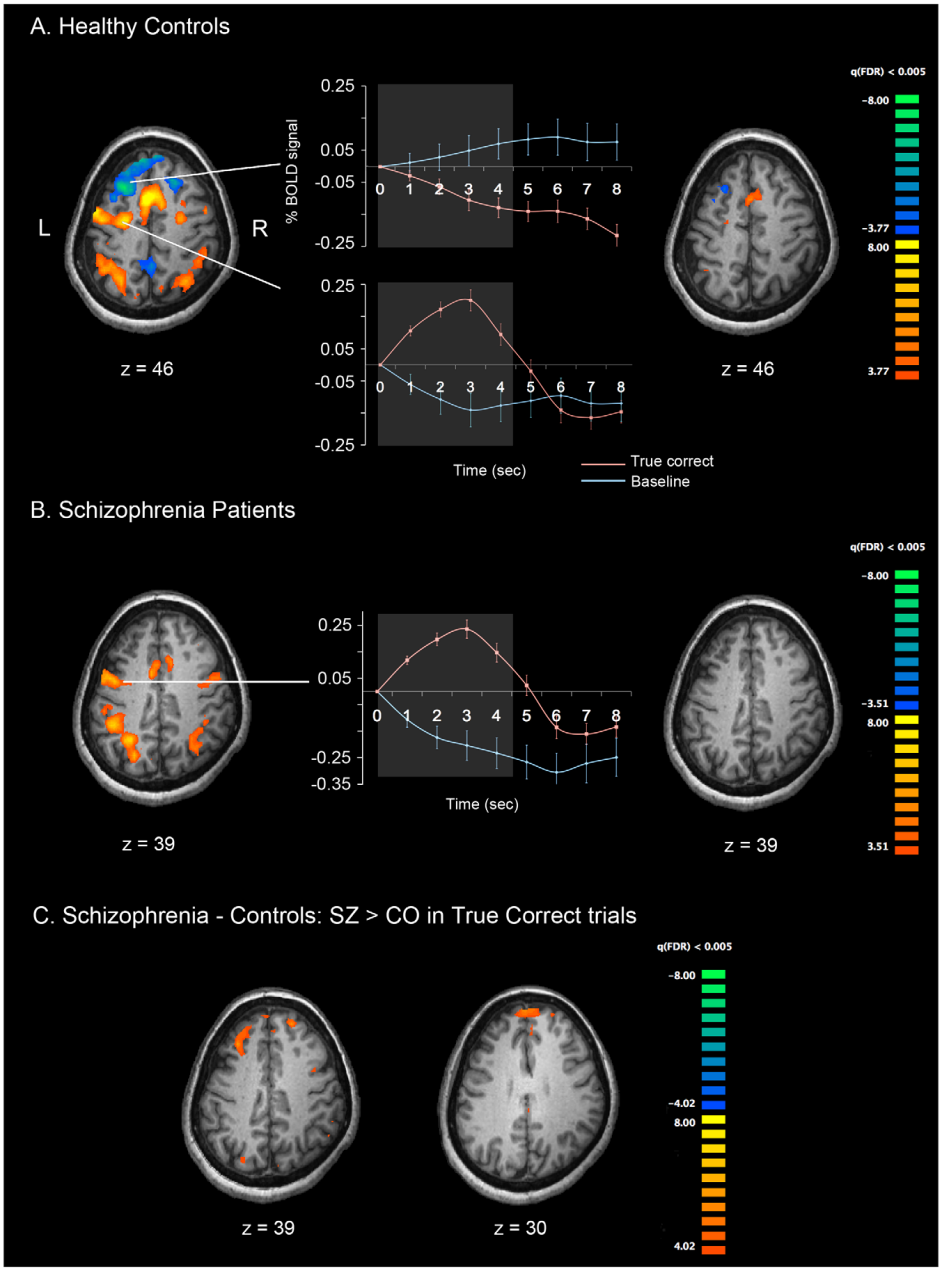
Cortical activation patterns during verbal WM maintenance for the two groups. Healthy controls (A), patients with schizophrenia (B), and significantly different activation between groups (subtraction of SZ-CO) (C) are shown. The time series plots in the middle column show activation associated with true memory maintenance (red lines) relative to the baseline activities (blue line). Bright parts in the middle of each plot represent 1-volume (1.5 s) after onset, and offset of the maintenance phase (4.5 secs). All p-values are corrected with false discovery rate of q<0.005.

To identify brain regions that were associated with phonological WM maintenance, we contrasted brain activity associated with true memory with baseline for each group, and then compared the activity between the two groups. Figures 2a and b (left) represent the activation patterns during the delay period for true correct memory trials in each group. Figure 2c represents the areas significantly different between the two groups.

In true correct memory trials, CO showed increased bilateral activation in frontal regions including medial frontal (BA6), left superior frontal (BA6), middle frontal (BA6/10), precentral gyri, right middle frontal (BA6/9) and inferior frontal gyri (BA9). SZ showed bilateral activation in medial, middle frontal and precentral gyri (BA6). Parietal activation in superior and inferior parietal lobule (BA 7/40) that is involved in sensory processing was observed bilaterally in both groups.

Interestingly, CO also showed “deactivated (less activation than baseline)” frontal and posterior regions, which was not observed in SZ. This deactivation was greater in the left superior frontal gyrus (Fig. 2a left), resulting in relatively greater activity in SZ within this region (Fig. 2c). The regions activated during the delay for true memory trials are listed in Table 2.

**Table 2:** Activated areas during verbal WM maintenance on true correct trials.

	L/R	x	Y	z	*t*	*p*	*q(FDR)*	BA
**CO**		> Baseline				0.000856	0.005	
Superior Frontal Gyrus	L	−2	8	54	10.2			6
Middle Frontal Gyrus	L	−26	−7	47	6.98			6
	L	−32	43	21	6.51			10
Precentral Gyrus	L	−44	−4	46	6.89			6
Middle Frontal Gyrus	R	26	−5	46	5.6			6
	R	39	31	32	4.9			9
Inferior Frontal Gyrus	R	40	6	27	5.87			9
Inferior Parietal Lobule	L	−33	−49	37	8.89			40
Parietal Angular Gyrus	R	29	−56	37	8.62			39
Insula	L	−29	21	9	3.36			13
	R	30	23	8	7.56			13
**CO**		< Baseline				0.000856	0.005	
Superior Frontal Gyrus	L	−26	24	49	−7.31			8
	L	−13	48	38	−6.05			9
	L	−3	59	28	−8.06			9
	R	21	26	45	−6.06			8
Inferior Frontal Gyrus	R	45	34	9	−4.75			46
Parietal Precuneus	L	−10	−45	31	−6.66			31
	L	−2	−49	51	−4.8			7
Limbic Cingulate Gyrus	R	2	−45	30	−7.53			31
**SZ**		> Baseline				0.000401	0.005	
Medial Frontal Gyrus	L	−6	4	51	8.97			6
	L	−44	2	38	7.10			6
	R	47	4	41	4.55			6
Frontal Precentral Gyrus	L	−38	3	25	5.46			6
Inferior Parietal Lobule	L	−41	−39	38	6.91			40
Superior Parietal Lobule	L	−29	−54	38	7.02			7
Parietal Supramarginal Gyrus	R	39	−37	34	5.74			40
Parietal Angular Gyrus	R	27	−56	33	5.81			39
**Group Difference**		SZ > CO				0.000058	0.005	
Middle Frontal Gyrus	L	−31	30	41	5.2			8
Superior Frontal Gyrus	L	−6	57	31	4.7			9
	R	18	47	38	4.5			8

*Brodmann Area. x,y,z are the Talairach stereotaxic coordinates.

There were a large number of correct but not confident trials (guesses), therefore we looked at the activation patterns for these trials (Fig. 2a, right panel). With the same level of threshold (q(FDR)<0.005), both groups showed very little activity compared with the baseline. CO still had greater activation than baseline in the superior frontal gyrus (medial BA8) and deactivation in left BA8/9. Comparison between CO and SZ did not reveal significant activation difference overall.

#### Error trials

**Figure 3:**
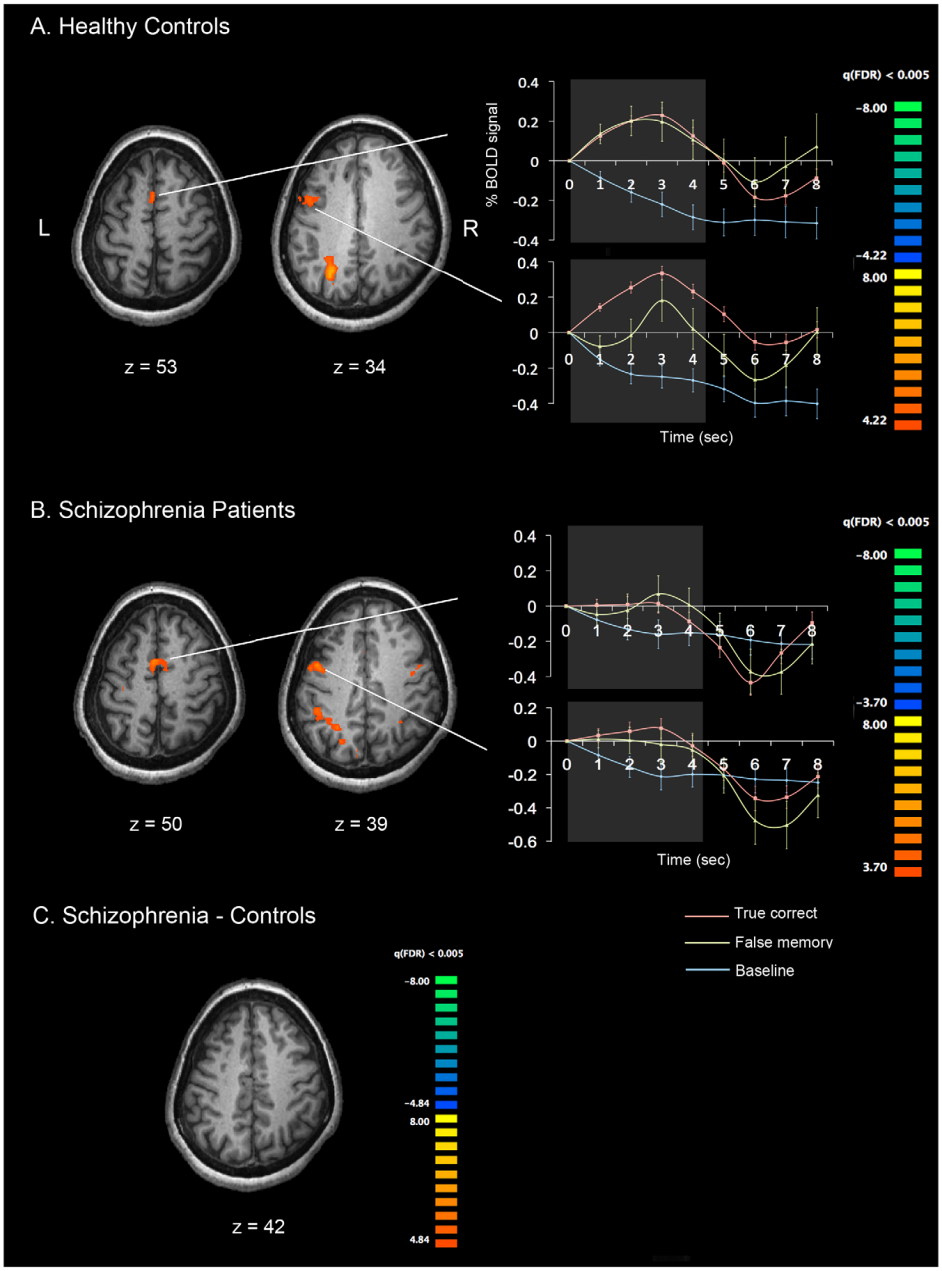
Cortical activation patterns during false memory trials. (A) False memory – Baseline in CO. (B) False memory – Baseline in SZ. (C) SZ – CO. All *p*-values are corrected with FDR of q<0.005. The time course plots show false memory related activities (yellow) and true memory related activities (red) relative to the baseline (blue).

BOLD activity during error trials with high confidence ratings were examined (see [13]). On false memory trials, both CO and SZ recruited only a subset of the regions that were activated in true memory trials, and significantly greater activation than baseline was observed mostly in left hemisphere (see Fig. 3). Unlike the true correct memory, CO did not exhibit “deactivated” regions on these false memory trials (Fig. 3a). We did not observe a significant group difference of frontal activation in false memory trials (Fig. 3c). The regions activated during the delay for false memory trials are listed in Table 3.

**Table 3:** Activated areas during verbal WM maintenance on confident but incorrect trials (false memory trials).

	L/R	x	y	z	*t*	*p*	*q(FDR)*	BA
**CO**		False Memory > Baseline				0.000025	0.005	
Superior Frontal Gyrus	L	−3	7	53	4.69			6
Middle Frontal Gyrus	L	−25−48	−95	4434	4.764.34			66
Insula	L	−30	19	8	4.93			13
	R	29	24	3	4.64			13
Parietal Angular Gyrus	L	−26	−57	34	6.33			39
Superior Parietal Lobule	R	29	−60	42	4.81			7
**SZ**		False Memory > Baseline				0.000069	0.005	
Medial Frontal Gyrus	L	−5	3	50	5.11			6
Middle Frontal Gyrus	L	−43	1	37	4.14			6
Inferior Parietal Lobule	L	−36	−48	41	4.31			40

We also asked whether greater activation is associated with the maintenance of incorrectly encoded internal representations (i.e., false memory) than simple guesses, we compared false memory trials with correct guess trials (figures are not shown). In SZ, greater activation for false memory was observed in right superior frontal gyrus (BA 9 (28, 43, 28), t = 4.71) and bilateral parietal regions (BA 7 (L: −14, −51, 50; R: 10, −57, 48), BA 40 (37, −35, 55), t = 4.05) at the q(FDR)<0.05. When we applied higher threshold (<0.005) used for the other analyses, this difference disappeared. In CO, there was no significant activation difference between these two trial types.

As for the true error trials (error trials with no confidence), CO had greater activation than baseline in the same area of the superior frontal gyrus (medial BA 8) that was activated for correct guesses (see Fig. 2a right). SZ showed no significantly activated regions at q(FDR)<0.005. As shown in Table 2, there were not many true error trials in both groups.

## Discussion

The present study investigated the brain activation pattern during WM maintenance associated with correct and error trials of phonological WM in healthy individuals and patients with schizophrenia.

Overall accuracy, when confidence ratings are not taken into account, indicated that our group of schizophrenic patients did not show a significant verbal WM deficit compared with CO. However, when we further examined how correct and error trials arose by analyzing different trial types, interesting differences emerged. Correct trials with low confidence ratings are likely to be guesses. We had hypothesized that guess trials would not correspond to changes in cortical activity above baseline because no WM maintenance is expected to have occurred. Overall accuracy score, when confidence ratings are not taken into account, included both true correct trials and guess trials. Therefore, it does not accurately reflect true accuracy of memory especially if there are many lucky guesses. When only ‘true memory’ trials (i.e. correct and confident) were considered, the group difference emerged, which suggests that SZ may be impaired in the phonological verbal WM task.

On the other hand, behavioral performance was not different in false memory trials between the two groups and this was also true for brain activation during these trials (Fig. 3). This finding diverges from the previous study of spatial WM by Lee et al. [13], in which they found increased rate of false memory trials in schizophrenia. This difference may be due to differences in task difficulty and available strategies for spatial and verbal WM tasks.

We had also hypothesized a reduced hemispheric asymmetry associated with correct trials in SZ based on previous studies [13], [33], [34]. In the present study, we did not observe reduced asymmetry in SZ compared with CO. CO showed bilateral activation in frontal regions, including the left superior and the middle, precentral gyri, the right inferior frontal gyri, and bilateral parietal regions on true correct memory trials. CO also showed regions of deactivation (relative to baseline activity) in the frontal cortex, including the superior and inferior gyri, the precuneus, and the cingulate gyrus in the left hemisphere. SZ also showed activation in regions of medial frontal, middle frontal, precentral gyri, and bilateral parietal areas. In error trials with high confidence (false memory), both CO and SZ showed more left-hemisphere lateralized activation pattern. Those activated regions overlapped with the regions activated in true memory trials. However, there was one important difference between the SZ and CO; CO did not show regions of deactivation on false memory trials that were observed on true memory trials.

Overall, the results of the present study suggest that verbal WM impairment in SZ cannot be simply described as either a problem of hyperfrontality or hypofrontality. Past studies have also reported discrepant findings on this issue, depending on the task difficulty and/or performance. For example, CO exhibited increasing DLPFC activation as performance decreased while SZ had the opposite pattern in a verbal WM task [25]. A meta-analysis also indicates a complex pattern of hyper and hypoactivation in schizophrenia [5]. In group comparison of the present study, SZ exhibited greater activation than CO in superior frontal areas (Fig. 2c) but this ‘hyperfrontality’ was due to deactivation relative to the baseline in CO rather than an increased activation in SZ. The results from false memory trials suggest that sometimes both CO and SZ maintain incorrectly encoded internal representation with corresponding cortical activation.

Healthy control participants appear to recruit different neural networks for maintaining items in verbal WM in true memory compared with false memory trials as indicated by deactivated prefrontal regions in true memory. Furthermore, the pattern and extent of activation is more bilateral and increased in true memory trials, whereas it is shifted leftward in false memory. Thus, CO seems to recruit a wider network during the maintenance of correctly encoded information. This was also true for SZ; patients also showed greater and less lateralized activation in true memory compared with false memory trials (Fig. 2b left and Fig. 3b). Unlike CO, however, SZ did not have deactivation relative to baseline in prefrontal regions.

Therefore, the most evident difference in activation patterns between groups related with our task is whether the superior prefrontal area (BA 8/9) was deactivated relative to baseline. However, ‘deactivation’ for correct memory in CO is not easy to explain and should be interpreted cautiously. On the basis of the results from CO in true memory trials, it is possible to assume that the activated frontal/parietal areas and deactivated prefrontal areas comprise or would be parts of a fully functioning network for verbal working memory. The activated areas in false memory would be also parts of the network (in fact, these areas overlap). Since deactivated areas were observed in correct trials only (note that there is also deactivation in correct guess trials), this deactivation is likely to be involved in maintaining correctly encoded internal representation. Therefore, this task-induced deactivation may reflect beneficial processes, for example, efficient reallocation of resources from default to task-relevant processes [26], [27], associated with correctly encoded information rather than reflecting detrimental processes [28]. Considering lack of such functionally relevant deactivation in SZ group and in false memory trials in CO, less task-induced deactivation in the prefrontal area during maintenance may have contributed to maintaining false representations. However, we do not argue that this deactivation is entirely responsible for maintenance of correctly encoded information because SZ did not show such deactivation even in true correct trials. At least, it is tempting to speculate that prefrontal deactivation would be beneficial for maintenance of correct information.

With respect to the activation pattern difference between true and false memory trials (i.e. bilateral vs. left-lateralized activation), it is worth noting that the participants had to phonologically decode visually presented stimuli during WM encoding in our task. It is hypothesized that during maintenance period, internal representations of the stimuli were supported. In our experiment design, we tried to minimize visual perceptual influences that could be used for encoding and retrieval. That is, if both the target and probe words were shown in identical cases or fonts, it may be possible to make a correct response by exclusively using the visual information (e.g. identical shape, font, or size). By making sure that the target and probe words were presented in different cases, we were trying to minimize the visual perceptual influence and the use of “visual features”, and to maximize the potential for phonological processing. However, our manipulation does not eliminate visual coding. Therefore, it would be more accurate to suppose that subjects had access to both visual and phonological representations that were maintained during true correct trials. During the retrieval stage, phonological-visual transformation must occur again because the probe word is visual. This effort may be reflected in a more bilateral activation pattern. Phonological decoding (grapheme-to-phoneme conversion) involves a network of the anterior left precentral gyrus and the left ventral occipitotemporal cortex [29]. One can maintain visual as well as the phonological representation of the nonsense words during the delay. Therefore, the bilateral activity observed in true correct trials might reflect this dual strategy. Activation in right parietal regions, which is involved in maintaining spatial and object information and possibly in WM manipulation [30]–[32], may also reflect active processing of visuospatial information during maintenance. Dual coding of stimuli and maintenance of both visual and phonological features could increase accuracy.

On false memory trials, the activation pattern was more left-lateralized. This may mean that what was maintained during false memory trials was probably phonological and perhaps the locus of the error lies in grapheme-phoneme conversion during encoding.

In the context of laterality, Lee et al. [13] found that CO had a right hemisphere advantage for processing visuospatial information while SZ exhibited more symmetrical activation pattern. Other studies also reported reduced or reversed hemispheric asymmetry in schizophrenia [33], [34]. In verbal domain, one might expect that CO would exhibit more left lateralized activation [35], [36] while SZ would have reduced asymmetry [22], [33]. Past studies have suggested that the lateralized activation in CO may reflect efficient and specialized processing and reduced asymmetry in SZ may indicate their inefficient and/or compensatory mechanisms [14], [22], [33].

However, other studies found bilateral activation for both verbal and spatial WM tasks [37]–[39]. A recent fMRI study [40] also suggested that a common bilateral frontoparietal network subserves both verbal and spatial domains but recruits additional left-lateralized frontal and temporal regions for further verbal processing. These studies suggest that the activation pattern of the frontoparietal network is shaped more by the task demands (manipulation, maintenance and/or both), and task difficulty than by laterality. Our data suggest that both CO and SZ recruit wider bilateral network of task-relevant brain areas perhaps reflecting the dual strategy to maintain true correct memory. As discussed above, prefrontal deactivation in CO in true memory trials might be associated with correct maintenance.

There are limitations and caveats. First, all patients were taking antipsychotic medication at the time of testing. Past results on the effect of antipsychotic medication on WM in schizophrenia are variable. For instance, atypical antipsychotic drugs appear to improve verbal and spatial WM performance in schizophrenia [41]–[43]. Other studies argue that improved performance on tasks after treatment is due to learning and practice rather than medication effect [44], [45]. Our SZ subjects did not perform significantly worse than CO overall, suggesting that medication effect may not be a critical confounding factor in interpreting our data. In addition, we examined correct and error trials separately, so the performance, by definition, was matched between the two groups. Second, our sample size is on the small side. However, the effect sizes were robust. We used a very conservative statistical criterion, i.e. very low false discovery rate of <0.005 to find difference between conditions or between groups in activation maps. Third, our primary purpose was to investigate brain activation related with verbal, phonological working memory. However, we had to extend discussion into visual domain because our task was not purely verbal by visually presenting verbal information. Comparing our results with future data collected by auditory presentation could reveal activation difference in processing visual-verbal and auditory-verbal working memory.

To summarize, we observed different patterns of brain activation in maintaining true memory and false memory in both CO and SZ: a wider frontoparietal network was recruited to maintain correctly encoded internal representation compared with maintenance of incorrectly encoded information. We found a subtle group difference in activation patterns in our study. CO showed prefrontal deactivation relative to resting activation in correct memory trials. Perhaps, a lack of such task-induced deactivation in schizophrenia may correspond to false memory.

Overall, these findings underscore the utility of parsing out different sources of WM errors to investigate accompanying brain activation and more broadly, the importance of elucidating the non-linear nature of brain-behavior relationship.

## Materials and Methods

### Participants

Twelve outpatients with chronic schizophrenia (SZ) were recruited from two private psychiatric facilities in Nashville, TN. The patients met the DSM-IV criteria for schizophrenia or schizoaffective disorder, based on structured clinical interviews (SCID) and chart reviews [46]. Clinical symptoms were evaluated using the Brief Psychiatric Rating Scale (BPRS) [47], the Scale for the Assessment of Negative Symptoms (SANS) [48], and the Scale for the Assessment of Positive Symptoms (SAPS) [49]. All patients were taking atypical antipsychotic drugs (clozapine, risperidone, or olanzapine) at the time of testing. Thirteen healthy control participants (CO) were recruited through advertisements in Nashville, TN. The two groups were matched in age, education level, handedness and IQ (See Table 4). All of the participants were native English speakers. No one had past or current substance abuse, head injury, neurological disease or medical illness affecting brain function. No CO had DSM-IV Axis I or II disorder, or a family history of psychotic disorders.

**Table 4:** Demographic information of the participants.

	SZ (n = 12; 5 women)	CO (n = 13; 5 women)	*p*
Age	40.2 (10.23)*	40.4 (9.34)	0.96
IQ (WASI)	92.0 (19.8)	99.3 (17.9)	0.39
Years of Education	14.1 (2.0)	15.4 (3.12)	0.23
Illness Duration (years)	14.1 (9.9)	-	-
BPRS	13.75 (6.6)	-	-
SAPS	10.0 (7.8)	-	-
SANS	15.5 (10.1)	-	-
Handedness (Edinburgh)	+58.8 (64.49)	+77.75 (20.44)	0.34

*Mean (standard deviation).

### Ethics Statement

Written informed consent was obtained from all participants after they were given a complete description of the study. The Institutional Review Board of Vanderbilt University approved the protocol and consent procedure.

### Phonological Verbal WM task

Functional images were obtained while participants performed a phonological delayed-response task (Fig. 1). At the beginning of each trial, a fixation cross was presented for 1 s. Then three nonsense words were presented in black on a gray background, each in a different location for 3 s. The stimuli were Dutch words between 4–6 letters and were phonologically similar to English words but were meaningless to non-Dutch speakers. Subjects were asked to silently read these stimuli. A delay period of 6 s followed. After the delay, a probe nonsense word was presented for 2.5 s and participants were asked to decide whether the probe was the same as one of the three target words by pressing one of the two assigned buttons. To minimize potential visual influence and visual strategy based on identical shape, font, or size on the screen, the probe nonsense word was presented in lower case if the targets were in upper case and vice versa.

After making the memory response, participants were given 2.5 s to indicate their confidence level of the memory response that they had just given, on a 3-point rating scale.

The inter-trial interval was 8.25 s and subjects completed 4 runs containing 27 trials per run. Each run had 5 pseudo trials (fixation only) and BOLD signals associated with these trials were regarded as baseline. The first and the last trials in each run were discarded prior to analysis for MR saturation. The trials with missing WM response and/or missing confidence rating were also discarded. Therefore, the total number of trials included in analyses varied across individuals.

### Image acquisition

All brain images were collected on a 3-Tesla Phillips Intera Achieva system with a birdcage head coil at Vanderbilt University Medical Center, Nashville, TN. Twenty five T1-weighted anatomical images parallel to the AC-PC line were acquired with T2*-weighted functional images for BOLD-based images, using echoplanar (EPI: TR = 1500 ms, matrix = 128×128, slice thickness = 4.5 mm, slice gap = 0.4 mm, FOV = 240×240 mm) sequence. High-resolution T1-weighted anatomical volumes were also acquired with a T1 3D turbo field echo (T1TFE) sequence (TR = 8.877 ms, matrix = 256×256, slice thickness = 1 mm, gap = 0 mm, number of slices = 170).

### fMRI data analysis

The imaging data were preprocessed and analyzed using Brain Voyager QX 1.10.2 (Brain Innovation, Maastricht, The Netherlands). Anatomical volumes were transformed into a common stereotaxic space [50]. Functional volumes for each subject were aligned to the anatomical volumes, thereby transforming the functional data into a common brain space across participants. Data pre-processing included image alignment, three-dimensional motion correction, linear de-trending, temporal frequency filtering with high pass filter, slice-time correction, and spatial smoothing with 4 mm Gaussian kernel (FWHM). Statistical analysis was based on the application of the multi-study general linear model (GLM) to the time-series of task-related functional volumes. A GLM with predictors of interest (i.e. correct vs. incorrect trials with/without confidence from behavioral data) was applied for the individual z-normalized volume time courses. To reduce possible mixture of signals from encoding and maintenance phases, the BOLD signals coupled with the latter 3TR (4.5 s) of the delay period were analyzed as WM maintenance activity. Significant difference among the conditions was assessed with contrast (t) maps at a false discovery rate (FDR) of q<0.005, using random effects statistical parametric maps (SPM).
